# Choroiditis in Relapsing Fever

**Published:** 1880-12

**Authors:** Julius Trompetter, Lucien Howe


### Translations.

### CHOROIDITIS IN RELAPSING FEVER, BY DR. JULIUS TROMPETTER.

FROM THE GERMAN BY LUCIEN HOWE, M. D.

ALTHOUGH the appearance of Choroiditis as a result of relapsing fever has been in former years occasionally observed in Breslau, still, we have so frequently had an opportunity, during the last epidemic, of noticing its course that we venture to offer the following observations as being of some interest to corroborate former statements, and to call attention to a few facts thus far unknown.

In the first place, the percentage of eyes affected during the last epidemic, could be determined with considerable exactness from the number admitted to the general hospital there. Dr. Spitz, who made this, the subject of an inaugural address, states that he observed five cases of choroiditis among 325 patients. In our department of the clinic, there were twelve cases treated, and four more seen otherwise, or 21 cases among 325, that is to say, about six per cent. This percentage is rather larger than that given by other writers, for example, by Blessig, who found in St. Petersburg, in 1864, only two per cent. of this trouble. Our figures also show a proportionately large number of men affected, but this is probably due simply to the fact that more of that sex happened to have the fever.

The ages of the patients ranged in general from 20 to 30, although one was only seven years old. Concerning the stage of incubation of the disease, that is, the time from the beginning of the fever to the appearance of inflammatory symptoms in the choroid—one can only arrive at a definite conclusion, by counting from the day when the last attack commenced. It is characteristic of this fever to vary in such a manner that statements of the patients in regard to its entire duration cannot be relied upon.

It was found thus, that choroiditis began in the first week after the last attack in two cases, in the second week after the last attack in four cases, in the third week after the last attack in four cases, and in the second month after the last attack in two cases.

I did not happen to see its development during the fever inasmuch as the patients presented themselves after having been once discharged from the hospital, but in the five cases reported by Spitz, it appeared in the progress of the fever. The eye symptoms referred to were of a decided inflammatory character. The amaurotic stage mentioned by earlier writers, could not be diagnosed because the patients applied for relief only after the trouble was well advanced, but several mentioned that for some days previously, they could not see in all directions as formerly.

Most of them, when first seen, presented the typical picture of a choroiditis, or more especially of cyclitis, which is well known by several characteristics.

In the first place the decided redness of the sclerotic, covered not only the portion near the cornea, but extended over the entire outer part of the globe. The tint of the sclerotic in the form of choroiditis is different from that shown in a simple iritis, and should not be forgotten in the differential diagnosis.

A second distinctive feature was the frequent appearance of hypopyon, although the iris did not seem to be involved. This was noticed in ten of the cases almost from the first, it showed itself in the form of fine yellow lines at the lower part of the anterior chamber, but never in any very great degree, and disappeared under proper treatment in a few days. Meanwhile, however, the iris preserved its normal color, the pupil reacted promptly and could be dilated to the fullest extent, by means of atropia, within an hour, and in only three cases was any deposit left on the anterior face of the capsule of the lens. Among these cases, I never saw that chronic form of choroiditis, which accompanies a sort of irritable iritis and which has been described by Logetschnikow as the asthenic variety.

A third characteristic of this form of choroiditis, was the opacity of the vitreous humor. It was noticed in every instance but has been so carefully described by other writers, especially *Estlander and Blessig, that it can here be passed over with simple mention.

The subjective symptoms of the disease, however, were of interest as offering a field for original study, especially in reference to the relation between the acuteness of vision and perception of light. The former, at the beginning of the trouble was usually so affected that in consequence of the floating particles in the vitreous, it was hardly possible for the patients to count fingers at a few feet distant. But as the swelling about the optic nerve subsided, and in general the fundus of the eye appeared clearer, when seen with the ophthalmoscope, then the vision began daily to improve even though some of the opacities in the vitreous persisted.

[The writer then goes into detail concerning certain peculiarities of the perception of light, and the contraction of the field of vision and thus continues.]

The progress is usually favorable, there being only one case among the number which resulted in a closure of the pupil. From patients under constant observation a good issue should be expected; but one who was treated in the out-door depart ment only improved slowly, from the adverse circumstances to which he was subjected. The first appearances of improvement were in the lessening of the injection about the cornea. Next there came gradually an absorption of the particles in the vitreous humor. in such a way that the small, diffuse opacities formed themselves into films, more or less dense and of irregular size, and finally sank in the lower portion of the globe. No permanent changes in the appearance of the choroid, nor exudates, nor partial atrophies could be seen.

The duration of the disease up to the time of the complete subsidence of inflammatory symptoms and return of normal

* Estlander on choroiditis after recurrent typhoid fever. Archives of Ophthalmology, vol. xv, part 2.

vision, was, on the average, from a month to a month and a half, although in the second and third month, even with perfect vision, a few opacities in the vitreous humor could be noticed. There was no relapse of the choroiditis, but in two cases it showed itself in both eyes. In both of these instances the attack was sudden in the second eye, and since these examples were worthy of study as illustrating the history of the disease, it may be worth while to refer to one briefly.

A. Ch., 48 years old, a trusty servant in the General Hospital here, was taken with relapsing fever in October of last year, and presented herself, fourteen days after the last attack, at the out-door department connected with the institution. The left eye had evident symptoms of an acute cyclitis (choroiditis), viz., injection about the cornea, hypopyum, and opacities of the vitreous humor. The patient applied atropine for several days, taking corrosive sublimate internally, still she refused to resign her place in the surgical department, and therefore the inflammatory symptoms persisted longer than usual. On the 26th of November the right eye was still perfectly good, and on the evening of that day the patient was occupied till quite late, sewing and reading. The next morning she noticed, immediately on awakening, that the vision of that eye was decidedly affected, and an examination then made revealed numerous particles floating in the vitreous humor. The second case in which choroiditis appeared in the other eye showed the same intensity of the symptoms, but no other peculiarity which would make it worthy of especial mention. It seems, therefore, that the choroiditis which comes after relapsing fever, develops in a comparatively very short time.

In view of the observations which have been made, there can be no further doubt of the relation of relapsing fever with choroiditis, the only question is as to how to explain this connection. Estlander considers the process as due to a faulty nutrition of the eye, which is brought about by the condition of the blood, found in this fever. Blessig thinks that the weakened action of the heart, so noticable after relapsing fever, giving rise to thrombi in the region of the choroid, may be the cause, or that it may depend upon the emboli which result from partial necrosis, or abscesses of the spleen. The last theory is at least supported by the acute character of the inflammation. For let us suppose that there could be such a process going on in the spleen (which during the last epidemic was almost invariably found), which could persist even after the termination of the fever; it is then not difficult to imagine that small particles could clog the capillary network of the choroid, and in this way produce a sudden impairment of vision.

The treatment was simple in all the cases; during the first few days atropine was frequently dropped into the eye, in order to produce a wide dilatation of the pupil, and at the same time small doses of mercury were administered internally. After that the occasional use of atropine alone was sufficient, and finally gentle cartharsis, with a view to the clearing up of the vitreous humor.—*Klinische Monatsblaetter fuer Augenheilkunde.*

